# Some Practical Suggestions for Mitigation of Psychological Burden of Hospitalized Patients with COVID-19

**DOI:** 10.30476/ijcbnm.2020.86819.1371

**Published:** 2021-01

**Authors:** Maryam Shaygan, Hamidreza Hassanipour, Maryam Mollaie

**Affiliations:** 1 Community Based Psychiatric Care Research Center, Department of Nursing and Midwifery, Shiraz University of Medical Sciences, Shiraz, Iran; 2 Student Research Committee, School of Medicine, Shiraz University of Medical Sciences, Shiraz, Iran

**DEAR EDITOR**

The coronavirus disease 2019 (COVID-19) outbreak has brought to people all over the world, in particular to patients, not only the risk of death, but also too much psychological pressure. Individuals who are quarantined at hospitals or homes might experience various adverse feelings. The potential lethality of the illness, some clinical symptoms of patients including shortness of breath and hypoxia, and devastating and sensational news, especially on social networks could lead to anxiety and fear in patients. Patients may also have feelings of guilt about potentially exposing their family members to infection. “Medical mistrust” which refers to lack of trust in medical treatments may also increase the patients’ overall sense of worry and despair. Inadequate information about the disease and insufficient transparency from authorities about the disease status could also make the patients feel anger as we can see in previous studies on outbreaks such as Ebola. ^1^ Reduced contact with others, especially loved ones, leads to boredom, frustration, and a sense of loneliness and isolation, which is distressing to patients. Based on clinical observations and patients’ reports, worry about the health of self and that of significant others, loneliness, and lack of entertainment are the most concerns of patients quarantined at hospitals or homes. Therefore, mental health services should provide mental health care for individuals who are quarantined.

On the other hand, fast transmission of virus restricts face-to-face psychological interventions. Moreover, due to heavy workloads and lack of standardized training in psychiatry or clinical psychology, mental health providers do not always know how to mitigate the psychological distress of patients. ^2^ It would be worthwhile to develop online or smartphone-based psychoeducation to promote mental health among patients quarantined for COVID-19. Psychologists, psychiatrists, and psychiatric nurses could use the Internet and online social networks, including WhatsApp and Telegram, to share strategies by reducing stress, frustration, and sense of loneliness and isolation in patients. 

During the outbreak of COVID-19 in Iran, psychological services have been widely established by mental health professionals in universities and academic societies. An example of such psychological services provided by Shiraz University of Medical Sciences is psychoeducational services via WhatsApp (PSW). PSW includes various intervention strategies to minimize the potential mental health problems that might arise among individuals who are quarantined during the COVID-19 pandemic. It offers a package of interventions through instant messaging in order to offer self-help guidance to patients. PSW provides cognitive therapy through educational video clips, podcasts, texts, and audio files to teach patients to know their cognitive biases towards their disease and the likelihood of adverse events occurring to them. Furthermore, mindfulness techniques are recommended to help patients recognize their negative thoughts and emotions about the disease and reduce the intensity and impact of those thoughts and emotions on their level of stress. With these techniques, the patients are taught to try to allow any emotion they feel to just be without striving to change it or push it away. ^3^ Relaxation techniques such as diaphragmatic breathing, progressive muscle relaxation, and imagination exercises are taught to patients using video clips and audio files. Patients are encouraged every day to practice these techniques and provide feedback on which techniques work best for them as well as adapt to their condition. The patients are informed that they don’t need to do all the techniques every day. Instead, they can choose the most effective technique for themselves and practice it daily. 

To target engagement, pleasure, and meaningful life for the management of hopelessness in such patients, positive psychotherapy exercises including “Positive Reminiscence”, “Three Good Things”, and “Hope, Optimism, and Posttraumatic Growth” exercises are provided. During the “Positive Reminiscence Exercise”, patients should take time to think about an event from their past that evokes positive emotions, visualize the event in detail, and focus on the pleasant feelings arising during the exercise. ^4^ The objective of the “Three Good Things” exercise is to help patients be more aware and remember the good things and positive events that happened to them throughout the day.5 During the “Hope, Optimism, and Posttraumatic Growth” exercise, patients are encouraged to think about the times when important things were lost, but other opportunities opened. ^5^

In addition to psychoeducational interventions, every day, the number of recovered patients from COVID-19 is sent to patients to increase hope in them. Moreover, music, humor, and riddle are sent through WhatsApp in order to entertain patients during their hospitalization. 

Finally, we should consider the fact that individuals who experience COVID-19 and face quarantine have various psychological demands. Thus, mental health professionals should provide psychological interventions according to their interests and needs.
